# How do US corporations communicate interculturally with their Chinese stakeholders: Analysis of GM Company’s social media posts from the cultural value perspective

**DOI:** 10.1371/journal.pone.0292552

**Published:** 2023-10-05

**Authors:** Jin Xu, Ruijun Duan

**Affiliations:** 1 Intercultural Department, General Education College, Jinhua Polytechnic, Zhejiang, China; 2 Faculty of Humanity, The Hong Kong Polytechnic University, Hung Hom, Hong Kong; University of Malta, MALTA

## Abstract

Social Media is an important means of communication with audiences around the world. The purpose of this study was to explore whether GM—a famous US auto company adapts its US Cultural values to suit the prevalent cultural values of its Chinese stakeholders on Chinese social media. Content analysis was used to evaluate the cultural content of GM Company’s posts on Weibo and Twitter. Although influenced by the special features of the car industry, there is still enough evidence that the communication style of the US auto Company makes cultural adaption on Chinese social media, reflecting more Chinese prevalent cultural values.

## Introduction

Globalization accelerates cultural contacts, and cultural values play an important role in shaping people’s awareness, perception, and lifestyles [1]. If the targeted public identity within a cultural group is different from the cultural group identity of the corporation, then the communication between the corporation and the public would be intercultural in nature [2,3]. Cultural value is of great importance for corporations because cultural value among the public may lead communicators to conduct different communicative patterns [4,5]. The need to be equipped with cultural sensitivity and intercultural communication competence has been increasingly recognized by multinational corporations [2,6–8].

In the current age, social media offers an important means for enabling new forms of communication and increasing the interactivity between multinational corporations and stakeholders from different cultures [9], especially with the sharp increase in social media use over the past ten years. It’s crucial for multinational companies to take the chance and employ more effective cultural-related strategies to engage more potential international customers [6,10]. Then how do multinational corporations communicate effectively with the public from different cultural backgrounds on social media? Do they adapt their communication style to the consumers’ local cultural values? This research aims to study the intercultural communication of international corporations on social media from a cultural value perspective by looking into a specific corporation, GM Company, a US world-leading automobile corporation.

GM Company had learned the importance of culture and embraced cultural diversity in its global communication campaigns. They believed cultural awareness enabled managers to develop appropriate strategies and determine how to plan and organize in a specific international setting. Also, culture influences consumers’ behaviors on product use and purchasing, which in turn affects the marketing performance of companies.

General Motors Company (GM), has four core automobile brands, i.e. Buick, Cadillac, Chevrolet, and GMC., is a US multinational auto manufacturing company with its headquarters in the USA. It’s America’s largest auto manufacturer and also one of the largest worldwide. China has always been the largest single target market for GM for the past 5 years. In 2021, the number of vehicles that GM Corporation sold to Chinese consumers reached 2.9 million. Therefore, it’s significant to study how one of the world’s largest automakers, GM, communicates interculturally with its Chinese consumers.

To examine GM’s social media communication from an intercultural perspective, we have chosen to focus on its communication with Chinese stakeholders via Sina Weibo since Sina Weibo is one of the oldest and most popular social media platforms in China, with a huge number of registered users [11]. Its extensive user base makes it an ideal choice for examining the communication strategies of multinational corporations like GM Company, as it allows for access to a diverse and substantial audience. Intercultural communication involved “interaction among members of two or more distinct cultural groups” [12] and hoped to achieve the “mutual creation of meaning across cultures” [12]. Along this line, the communication between GM Company and its Chinese stakeholders via Sina Weibo could be regarded as intercultural since GM originated in the United States while the audience is China-based. For comparison, we have also included Twitter, a popular social media in the United States [13] which facilitates communication between the corporation and its audience in the United States. The comparability of Twitter with Weibo is proved by some research as it has a very similar function and characteristics to Weibo [14].

To be more specific, the research aim of this study is to explore whether GM Company adapts its US Cultural values to suit the prevalent cultural values of its Chinese stakeholders on social media. The cultural value framework proposed by Singh [15] is adopted to analyze the communication style influenced by local cultural values on GM Company’s social media. Thus, one contribution is that this study validates the cultural value framework by extending it to social media rather than only for advertising analysis on TV or websites.

## Literature review

### Corporation-stakeholder intercultural communication

Culture is defined by Hofstede as “the collective programming of the mind which distinguishes the members of one group from another” [16]. It is expected that within a given national culture there is a dominant set of shared values, attributes, beliefs, and behaviors. Therefore, intercultural communication, the communication between people from two different cultures, is a field of study that investigates how individuals from different cultural backgrounds strive to communicate across cultures [17]. Previous research indicates that multinational corporations’ competence and strategies of intercultural communication play a crucial role in marketing and attracting customers from different cultural backgrounds [18]. Appropriate intercultural communication not only helps multinational corporations deliver products and services that resonate with their target markets but also gains higher customer satisfaction and loyalty [19].

With the accelerated globalization of economic activities and the rapid development of new communication technologies over the past decade, there is a growing number of empirical studies on intercultural communication between corporations and their stakeholders from different cultural backgrounds for the last few decades [20,21]. For example, Matusitz [22] examined Disney’s successful experience in Hongkong and found that its success is due to its adaption to local labor practices, and adaptation to local visitors’ customs by changing decorations and settings. Another study [23] looked at the effect of culture on marketing communication through the websites of four multinational fast-food companies operating in the USA and Malaysia. This qualitative study used a content analysis design to assess the websites of four fast-food international restaurants namely: Burger King, KFC, McDonald’s, and Domino’s Pizza operating in both the USA and Malaysia. The objective of the study was to find out how cultural differences and various dimensions of culture affect the design of the websites of these multinational companies. The study revealed that the sampled websites reflected the local cultural values of the location of the various companies, indicating that making cultural adaptations helps to attract local customers.

Although much previous research revealed that international companies were practicing cultural adaptation when communicating with stakeholders from different cultural backgrounds, there are also some contradictory findings concerning corporates’ intercultural communication styles. Research by scholars like Singh et al. [24] suggests that the cultural elements and contents on local websites of India, China, Japan, and the US reflect the cultural values of the country of their origin rather than the customers’ cultural values. Research by Kim et al. [25] also indicates the significant impact of brands’ origin countries. What’s more, most of the research on corporations’ communication with stakeholders from different cultural backgrounds did not cover the aspect of social media communication, which is becoming increasingly important in brand communication as it enables brands to reach customers from all over the world more easily and faster [26]. Therefore, multinational corporations’ intercultural communication on social media still warrants further attention. However, there is even less literature focusing on social media communication in the auto industry. Due to the conflicting findings and deficiency in previous literature, we aim to find out in this research whether international auto corporates’ intercultural communication style on social media is largely based on the cultural values of its stakeholders or the cultural values of the company’s origin country.

### Cultural value framework

To study the corporations’ intercultural communication style on social media with stakeholders from the cultural value perspective, a theoretically grounded and empirically validated framework, which could provide a clear explanation of the cultural variation of different societies is in need.

At the very beginning of the globalization of business, Hofstede proposed a classic cultural value framework based on four dimensions: Individualism-collectivism, power distance, uncertainty avoidance, and masculinity-femininity [16]. He suggested this four-dimensional value concept of culture [16] after surveying around 117.000 employees of IBM in around 50 countries. His research was the foundational work for cultural value research in the business and communication field and created the basis for thousands of empirical articles [27–29]. Although Hofstede has expanded the four dimensions to six in his later research [30,31], the initial four dimensions remain the most classic and widely adopted framework [29].

However, there are still some other popular cultural value frameworks, such as Schwartz’s Value Survey and Trompenaars and Hampden-Turner’s Model of National Culture Differences [32]. Like Hofstede, Schwartz conducted large-scale self-report studies to investigate the phenomenon of culture across countries. Schwartz developed culture taxonomies and measurements for the individual [33,34] and country level [35] by surveying teachers and students from 44 countries and by surveying people from 63 countries [36]. Schwartz’s value framework is still one of the most common frameworks to examine cultural values. For instance, the European Social Survey [37] is based on Schwartz’s values and has been widely used by cross-cultural researchers [38–40]. One limitation of Schwartz’s value classification for cross-cultural business communication research is its low relation to the business and marketing context, leading often to weak effect sizes or non-significant results [38].

Another famous framework to measure culture is provided by Trompenaars and Hampden-Turner [32], who identified seven dimensions to classify cultural values. Trompenaars and Hampden-Turner surveyed over 30.000 people in around 30 countries to identify a value structure. They based their value category on the cultural framework of Kluckhohn and Strodtbeck [41]. The dimensions provide information about a) how people deal with each other, b) how they deal with time, and c) how they deal with their environment and nature. This framework is interesting, however, by comparing his framework with Hofstede’s dimensions, not much substantial differences are found. For example, the achieving orientation is similar to the masculinity orientation as both dimensions focus on the importance of achievement, success, and advancement. The being orientation is similar to the femininity orientation, and the hierarchy orientation is similar to power distance. Individualism and collectivism are included in both frameworks. Although the dimension of relation to the broad environment and nature of humans did not overlap with Hofstede’s framework, these two dimensions are not so closely related to the current topic of branding on social media.

Among all these frameworks, we adopted Singh et al.’s [42,43] cultural value framework for guiding our research on General Motors’ intercultural communication style on social media with its Chinese stakeholders. This framework complements the very classic Hofstede’s cultural dimensions with Hall’s high-low context theory (1990) [24]. Unlike some other models that may focus solely on national cultural dimensions, Singh and Matsuo’s framework was based not only on the four most classic cultural dimensions, power distance index, individualism-collectivism, masculinity-femininity and the uncertainty avoidance, proposed by Hofstede but also include the very influential high-context and low-context communication theory [44,45] in intercultural communication. High and low context theory provides a nuanced understanding of the implicit and explicit messaging prevalent in brand communication and advertising [46]. Considering that brand communication on social media communication involves a large amount of both explicit and implicit messages [47] and it could be challenging for a multinational auto company to decide how the information should be presented to people from different cultures, e.g. in an explicit or implicit manner. Therefore, this inclusion of high and low context theory is valuable for our research focus.

Meanwhile, Singh and Matsuo’s framework was not only theoretically grounded but also empirically validated in numerous previous studies to investigate cultural variations in communication [24,42,43,46], ensuring the validity of our research findings. Furthermore, this framework operationalizes those classic cultural dimensions proposed by Hofstede [16] and Hall [45] by offering a comprehensive set of sub-categories that capture the nuances of communication styles influenced by cultural values which is more operable than other general frameworks in analyzing social media posts [24]. Finally, the adaptability of Singh ’s framework to the social media context was a significant factor in its selection. With the growing significance of social media as a communication channel for multinational corporations [48], it was imperative for us to choose a framework that could effectively analyze cultural adaptations in the online environment. Singh and Matsuo’s framework, initially designed for the research purpose of online advertising, was well-suited to examine GM Company’s communication styles on social media platforms, thereby offering a lens through which we could explore GM’s intercultural communication strategies effectively.

### Hypothesis

As the main research objective is to examine whether US Corporation makes cultural adaptation in intercultural communication with their Chinese stakeholders on social media, this study makes a comparison of the GM company’s posts on Weibo and Twitter, attempting to see whether GM depicts more Chinese prevalent cultural values in Weibo than in Twitter. To examine this, five cultural dimensions of Singh [15,42], consisted of four dimensions of individualism-collectivism, uncertainty avoidance, power distance and masculinity-femininity from Hofstede [16] and one high-low cultural context dimension from Hall’s theory [45], were compared with the latest updated Chinese and American cultural value scores on these dimensions from *Hofstede Insight* [49] and Hall’s research [44]. In this way, three dimensions where Chinese and American has the greatest discrepancy, namely, individualism-collectivism dimension, power distance dimension high- and low-context cultures were identified.

#### Individualism-collectivism dimension

This dimension reflects how much society values group norms or individual liberty. Individualist culture-oriented societies place greater importance on personal freedom, personal accomplishment, and the freedom of individual decision-making, whereas collectivist culture-oriented societies tend to attach greater importance to obeying group norms, group achievement, and strong group connections and relationships. For example, in collectivist societies like China, sacrificing oneself for the benefit of the community or society is encouraged [50]. Meanwhile, in-group obligations, interdependence, and protecting others’ welfare are also emphasized [51]. It has been proved by previous studies that in a collectivist society, societal pressure and group norms have a very important impact on people’s behavioral formation. Therefore, advertisements in collectivist societies put much emphasis on group-consensus appeals, family ties, and family securities [52,53].

Individualist cultures encourage self-reliance, accomplishment, independence, and freedom since identity is based on “I-consciousness” (Hofstede, 2010). Individual determinism, independence, competitiveness, autonomy, and non-conformity have all been used in commercials in individualist societies to promote the independence theme [54]. According to *Hofstede Insight* [49], the US ranks 91 on individualism, whereas China scored only 20. Based on the scores, we hypothesize that:

*H 1 a GM Corporation communicates a higher level of collectivism-oriented features in Weibo posts than in Twitter posts*.

*H 1b GM Corporation communicates a lower level of individualism-oriented features on Weibo than on Twitter*.

#### Power distance dimension

The power distance cultural dimension, according to Hofstede et al. [55], describes how various cultures deal with social structural inequalities. High power distance cultures lay emphasis on referent power, social status, legitimacy, authority, and so on, whereas low power distance societies emphasize the value of equality (e.g. equal human rights) and less hierarchy. People in high-power-distance societies are more likely to respect authoritative figures. Chinese consumers, for example, are heavily impacted by opinion leaders and authority figures in their purchase decisions [56]. Based on past research that shows high-power-distance societies portray higher-power-distance-related appeals in commercials, such as status appeals, hierarchy, quality assurance and awards, the image of important people, etc, it’s reasonable to make the hypothesis that companies’ social media in posts high-power-distance societies’ will similarly show more high power distance features. According to *Hofstede Insight* [49], China scores 80 on power distance while US scores only 40. Thus, we hypothesize that:

*H 2 GM Corporation communicates more power distance features on Weibo posts than on Twitter posts*.

#### High- and low-context cultures

“A high context communication or message,” according to Hall [44], “is one in which most of the information is already in the individual, while very little is in the coded, explicit, communicated element of the message.” Harmony, beauty, and oneness with nature are valued in high-context civilizations [54]. Advertisements in high-context societies feature politeness in the use of language, using implicit and indirect messages and being polite and making friends with the customer rather than directly selling the products [57]. Low context communication is the opposite, in which the explicit code holds the majority of the information. Discounts, sales promotions, and aggressive selling approaches are typical in low-context cultures and the tone of communication in such cultures is direct and rhetorical in style [58]. Typical low-context cultures, such as the United States, make explicit mention of competitors’ products and use many hard-sell strategies [59]. Contrary to the communication in low-context cultures, communication style in high-context cultures, such as China, communication style is implicit, indirect and sentimental. According to Hall [44] and Hall and Mildred’s research [45], the United States has a low-context culture, while China has a high-context culture.

Therefore, the following hypothesis is generated:

*H 3 a*. *GM Corporation shows more high-context-oriented features in Weibo posts than in Twitter posts*.

*H 3 b*. *GM Corporation shows fewer low-context-oriented features in Weibo posts than in Twitter*.

### Methodology

#### Data collection

For the examination of communication between GM Company and its Chinese stakeholders, we selected the Sina Weibo accounts of four auto brands of GM Company, Cadillac, Buick, Chevrolet, and GMC, for data collection. Since popular social media accounts including Facebook, Twitter, and Twitter are banned from use in China, most multinational corporations have mainly relied on Sina Weibo, one of the most popular Chinese-based social media platforms in China with 516 million active online users in 2019 for communicating with their Chinese stakeholders [9,60]. For comparison, we have chosen Twitter, an English-based social media platform widely used in the United States [13] which largely facilitates communication between GM Company’s auto brands and its audience in the United States, very similar to and comparable to Sina Weibo [14].

We captured all posts (i.e., Chinese posts on Sina Weibo and English posts on Twitter) from November 1, 2021, to October 31, 2022. To avoid the issue of an unbalanced number of posts published from the two social media platforms, 120 posts on Weibo and 120 posts on Twitter were selected from the enormous database based on systematic random sampling.

### Compliance with terms and conditions

It is crucial to note that the data collection and analysis methods employed in this study adhered to the terms and conditions stipulated by the respective platforms. We strictly followed the guidelines provided by Sina Weibo and Twitter to access and analyze public posts for research purposes. Additionally, we ensured that the selected posts adhered to the platforms’ policies and did not involve any sensitive or private information.

### Theoretical framework, coding scheme and procedure

Content analysis was used to evaluate the cultural content of GM Company’s posts in Weibo and Twitter. Content analysis is a widely used method in communication studies [61]. Researchers could integrate a framework and adopt content analysis to identify the characteristics of a communication process [62], which is suitable for the current study.

**Fig 1:**
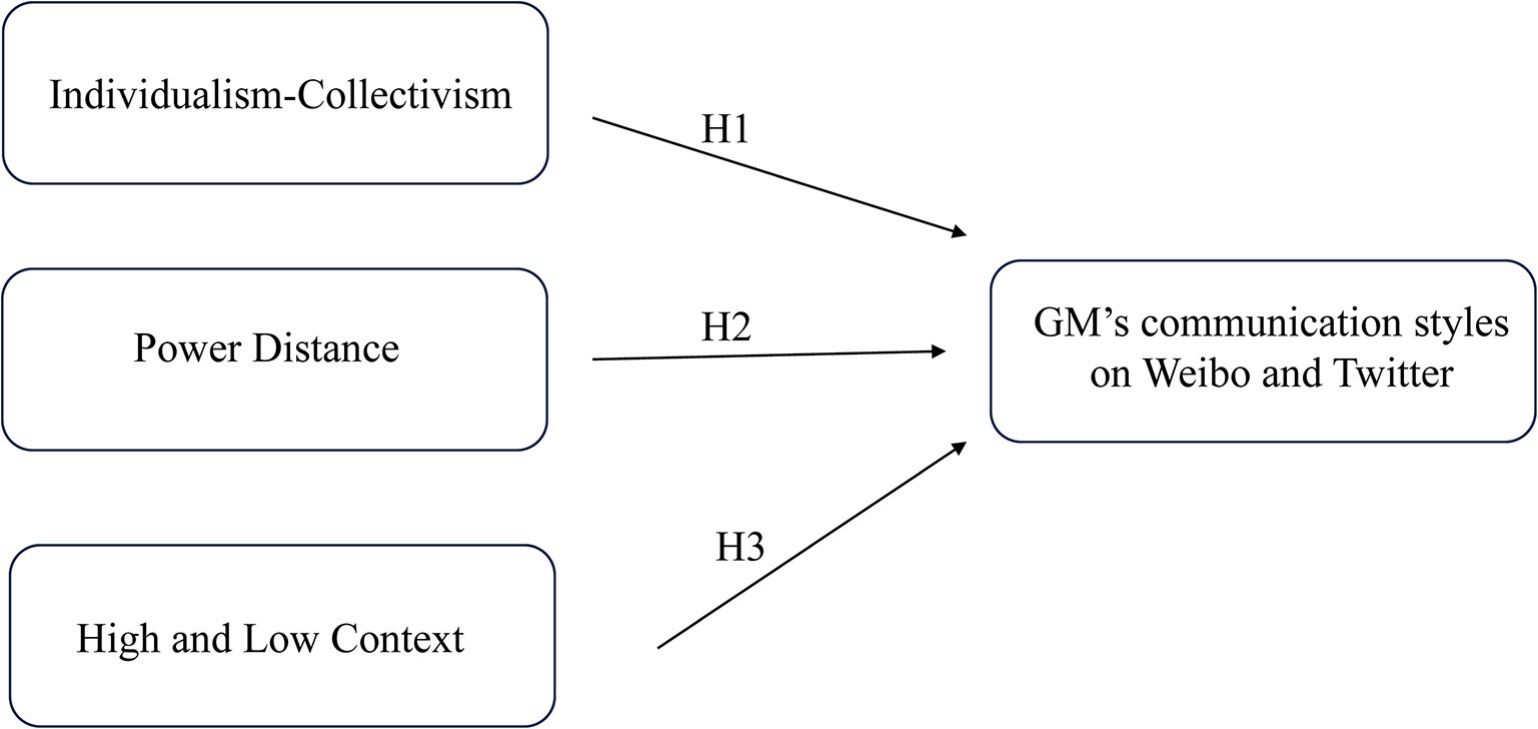
Conceptual framework. Note. adapted from Singh [15].

To develop an analytic framework for comparing the differences in communication styles on two different social media platforms, we adapted the cultural value framework from Singh’s research [15,42] which consisted of five dimensions with four dimensions from Hofstede [16] and one high-low context dimension from Hall and Hall [45]. As our research aim is to examine whether GM makes cultural adaptation on Chinese social media platform, it is necessary for us to choose the cultural dimensions that China and US has great disparity according to the latest updated Chinese and American cultural value scores from *Hofstede Insight* [49] and then investigate the effects of these three cultural values on GM’s communication styles (See Fig 1). An operational coding scheme with cultural dimensions, sub-categories, and descriptors was also adapted to investigate the GM’s social media communication styles in China (See Table 1).

**Table 1:** Cultural dimensions and sub-categories.

Cultural Dimensions	Sub-categories	Description
**Collectivism**	Community	*emphasis on community-based social order*
	Symbol	*the use of symbols and pictures of national identity*
	Family theme	*depiction of family integrity*
	Loyalty	*Building lasting relationships and engendering a sense of loyalty*
**Individualism**	*Independence*	*being independent; depicting self-reliance*, *freedom*, *self-sufficiency*, *and control over one’s life*.
	Goal-Orientation	*Striving for one’s own goals*, *desires*, *and achievements*
	Strong identity	*Knowing oneself*
**power distance**	Hierarchy	*hierarchical structures and stress coercive and referent power*
	Important people	*authority figures*
	Quality assurance and award	*certifications*, *awards*, *and prizes are viewed as symbols of universal recognition*
	Pride of ownership	*the emphasis on the status appeal*
**High context**	Soft-well Approach	*Emotions*, *sentiments*, *and entertainment themes*
	Politeness	*implicit and indirect messages*; *being polite and making friends with the customer rather than directly selling the products*
	Aesthetics	*emphasize harmony*, *beauty*, *and oneness with nature*
**Low context**	Terms and Conditions	*emphasizing clarity*, *background information*, *and directness*
	Rank and Prestige	*use comparisons to highlight the benefits and the prestige of the products and the company brand*
	Use of Superlative	*the tone of communication in such cultures is direct and rhetorical in style*
	Hard-sell Approach	*Being direct*, *explicit*, *and even confrontational; making explicit mention of competitor products and emphasizing sales orientation*.

Note. adapted from Singh [15,42].

The coding process was conducted by two coders. Necessary training on coding was provided to the two coders. Each cultural value dimension was rated from “not featured” to “prominently featured” on the five-point Likert Scale. To ensure inter-rater reliability in the coding, the two coders held two face-to-face meetings to reach a consensus on the coding scheme and procedure. Any confusion between the two coders was discussed in the coding process. The measure of inter-rater reliability was based on co-coding some of the posts from the two databases and 20 percent of the total number was studied. The average agreement was higher than 0.9, and the average Cohen’s Kappa was greater than 0.9, indicating an almost perfect agreement [63].

Then for the data analysis part, this study uses a t-test to analyze how GM company’s Weibo and Twitter posts differ in their communication styles from the cultural value perspective and also to measure the degree of cultural values on these two social media. The three cultural dimensions act as dependent variables, and the two social media, Weibo and Twitter act as independent variables.

## Results

The content analysis of GM Company’s posts on Weibo and Twitter revealed that overall speaking, there were significant differences in the depiction of cultural values between these two social media platforms. T-test and descriptive statistics were used to test the hypotheses about the differences between GM Company’s Weibo and Twitter platforms in their cultural values (Table 2).

**Table 2:** Descriptive statistics and T-test results.

Dimensions	Twitter		Weibo		T	P
	(n = 48)		(n = 48)			
	M	SD	M	SD		
Collectivism	3.181	0.811	3.354	0.521	-1.248	0.216
individualism	3.385	0.475	2.922	0.814	3.407	0.001
power distance	3.208	0.936	3.583	0.746	-2.171	0.033
High context	3.071	0.751	3.591	0.503	-4.042	0.000
Low Context	3.168	0.741	2.301	0.559	6.378	0.000

As shown in Table 2, posts on Weibo didn’t show significantly higher levels of collectivism (mean: Weibo: 3.354 vs. Twitter: 3.181, T = -1.248, P = 0.216). Thus, Hypothesis 1a was rejected. However, GM’s posts on Weibo showed fewer individualism features than Twitter (mean: Weibo: 2.922 vs. Twitter: 3.385, T = 3.407, P = 0.001). Hypothesis 1b was supported. For hypothesis2, GM Corporation’s posts on Weibo showed more power distance (mean: Weibo: 3.583 vs. Twitter: 3.208, T = -2.171, P = 0.033). Thus, hypothesis 2 was supported. Finally, for Hypothesis 3a, GM Corporation showed more high-context oriented features in Weibo posts (mean: Weibo: 3.591 vs. Twitter: 3.071, T = -4.042, P = 0.000), while Twitter posts were found to be higher on low-context dimension (means: Weibo: 2.301 vs. Twitter: 3.168, T = 6.378, P = 0.000), indicating that H3b was supported.

As mentioned above, except for Hypothesis 1a, all other hypotheses were supported. For further insight, we conducted a T-test on all the cultural categories (Table 3). The findings indicated that GM company’s Weibo and Twitter posts both scored high on collectivist features like symbols and family themes. For the cultural dimension of individualism, all the sub-dimensions of Twitter posts scored significantly higher than Weibo, i.e., independence, goal orientation, and strong personal identity. As for the power distance dimension, GM Company’s Weibo posts scored significantly higher on the sub-dimensions of hierarchy, quality assurance, and pride of ownership. As for the High and low-context cultural dimensions, Weibo posts scored considerably higher on the high-context dimension and sub-dimensions of politeness and aesthetics. Meanwhile, Twitter posts scored significantly higher on the low-context cultural dimension and the sub-dimension, e.g. rank and prestige and terms & conditions. Thus, a detailed cultural category level analysis clearly showed how the GM Company’s posts on the two social media platforms differ on each sub-dimension used in the framework.

**Table 3:** Descriptive statistics and T-test for cultural categories.

Categories	Twitter		Weibo			
	(n = 48)		(n = 48)		T	P
	M	SD	M	SD		
**Collectivism**						
Community	2.940	0.932	3.250	0.636	-1.919	0.058
Symbol	3.130	1.064	3.350	0.863	-1.159	0.249
Family theme	3.310	1.014	3.480	0.772	-0.906	0.367
Loyalty	3.100	0.928	3.230	0.660	-0.760	0.449
**Individualism**						
Independence	3.630	0.672	2.980	1.000	3.714	0.000
Goal-Orientation	3.330	0.808	2.900	0.973	2.397	0.019
Strong identity	3.330	0.724	2.880	1.003	2.567	0.012
**Power Distance**						
Hierarchy	3.170	1.260	3.710	1.031	-2.305	0.023
Important people	3.190	1.104	3.400	1.067	-0.940	0.350
Quality assurance and awards	3.270	1.125	3.710	0.849	-2.150	0.034
Pride of Ownership	3.210	1.031	3.520	0.743	-1.704	0.092
**High Context**						
Soft-sell Approach	3.120	1.022	3.660	0.645	-3.163	0.002
Politeness	3.020	0.896	3.340	0.713	-1.920	0.058
Aesthetics	3.170	0.923	3.610	0.784	-2.495	0.014
**Low Context**						
Terms and Conditions	2.841	1.256	2.654	1.101	0.778	0.439
Rank and Prestige	2.864	0.852	2.442	0.916	2.318	0.023
Use of Superlative	2.909	1.254	2.519	1.093	1.627	0.107
Hard-sell Approach	3.227	1.008	2.827	0.985	1.963	0.053

## Discussion

Based on the results in Table 3, the conclusion could be drawn that GM’s Weibo posts significantly differ from Twitter posts for most of the cultural value-influenced communication styles. For example, GM communicated less individualist cultural values on Weibo than on Twitter to suit the prevailing social values of China. Different from US’s individualist culture, China, heavily influenced by Confucian moral values and principles [64], has long been considered as a collectivist society where the value of interdependence and loyalty prevails. Although recent decades have seen some decrease in the value of collectivism, much research still indicates that it is a highly collectivist-oriented country [65–67]. People are not encouraged to have unique personal identities, instead, they are more encouraged to behave by certain societal norms. It is likely that GM may be disinclined to impose their cultural values of individualism onto their collectivistic Chinese-speaking customers, thereby leading them to downplay individualistic values on Weibo.

On the power distance cultural dimension, GM communicated this cultural value more prominently on Weibo than on Twitter. Cultural categories such as hierarchy, important people, quality assurance & awards, and pride of ownership are prominently depicted on Weibo. Because China is a high-power distance society that tends to show much respect to those authority figures. Chinese purchasing intention is easily influenced by the opinions of authority figures and leaders [68]. GM depicts more elements showing power distance, such as hierarchical structures, famous people, quality assurance and awards, and status appeal on its Weibo posts than Twitter posts, indicating its inclination to adapt to Chinese prevailing cultural values.

Compared with GM Company’s posts on Twitter, its posts on Weibo communicated more of a soft-sell approach which is more indirect in promoting its products and often used in a high-context culture. On the contrary, GM posts on Twitter were found to use a more hard-sell approach and communicate product information more directly and explicitly.

However, surprisingly, GM’s Weibo posts didn’t communicate collectivism significantly higher than Twitter although Chinese society is always considered to be collectivism oriented. GM communicates equally high levels of cultural values on both platforms. One possible explanation may be due to the unique characteristics of the car industry and the claim that advertisements sell dreams—-what they are longing for (Hood, 2005). Many auto advertisements tend to convey some family themes in their advertisements, creating a warm and harmonious picture of a big family sharing a car. Especially in an individualism-oriented society, like the US, auto brands try hard to sell such a dream. Therefore, GM pays much attention to communicating collectivism on both social media platforms.

In conclusion, enough evidence is provided from the results that GM corporation orients itself to the cultural values of the target society on social media platforms so as to appropriately position and market its products. This finding is congruent with some of the research in marketing that communication styles are heavily influenced by the target audience’s cultural values [8,14], but is contrary to other literature which indicates that international corporation’s communication style is influenced by the company’s origin country [24].

### Conclusion

This research aimed to explore how US corporations, particularly General Motors (GM) Company, communicate interculturally with their Chinese stakeholders on social media, focusing on the adaptation of cultural values. By analyzing the content of GM’s social media posts on Weibo and Twitter, we investigated whether the company aligns its communication style with the prevalent cultural values of its Chinese audience.

The findings of the study revealed significant differences in GM Company’s communication styles between Weibo and Twitter, indicating a strategic cultural adaptation by the corporation to engage its Chinese consumers. Firstly, in the dimension of individualism-collectivism, GM Company’s Weibo posts were found to communicate fewer individualism-oriented features compared to Twitter. This suggests that GM recognizes the importance of collectivism in the Chinese culture and tailors its messaging to promote group-oriented values and interdependence. Secondly, regarding the power distance dimension, GM’s Weibo posts depicted a higher level of power distance compared to Twitter. This finding reflects GM’s acknowledgment of the hierarchical nature of Chinese society and its emphasis on respecting authority figures and leaders in influencing purchase decisions. Furthermore, the communication style in high-context and low-context cultures differed significantly between Weibo and Twitter. GM’s Weibo posts demonstrated more high-context-oriented features, utilizing implicit language and emphasizing aesthetics and politeness. In contrast, GM’s Twitter posts had a higher emphasis on low-context communication, using direct language and explicit details.

Overall, the research results confirm that GM Corporation indeed does much adaptation in its communication style on Chinese social media to align with the cultural values of its Chinese stakeholders. By doing so, GM effectively establishes a stronger connection with its audience and better positions its products in the Chinese market.

### Implications and future research

This study benefits researchers and practitioners in many aspects. First, it broadens the scope of intercultural communication research by investigating the corporation’s communication styles influenced by cultural values. Despite the influence of the uniqueness of the car industry, the result still provides enough evidence that there are great differences in the communication style from the cultural value perspective between GM company’s Weibo and Twitter posts. Thus, we should have the awareness that social media is not always culturally neutral but is influenced by the consumers’ local cultural values. Second, GM company is a leading auto brand and enjoys a large market share in the Chinese car market, which could be considered a success in its communication with Chinese stakeholders. Therefore, it would give much enlightenment to other car companies in their communication with stakeholders from different cultural backgrounds. Lastly, this paper consolidates the cultural value framework developed by previous research and outlines the cultural categories and their explanation.

However, there are limitations to this study: First, the sample size of 120 posts on each social media platform may not be large enough to ensure the reliability of the results. Further research is needed to collect a larger sample of posts to ensure the generalizability of the results. Secondly, this study only examines the cultural value-influenced communication styles by looking into a specific auto corporation, so further studies are still required to investigate more corporations and even other industries. Furthermore, this study only focuses on two countries and three cultural dimensions, individualism-collectivism, power distance, and high and low context, where China and US have the greatest difference. Thus, future studies could also extend the scope to other countries and investigate other cultural dimensions.
